# MicroRNA858a antagonistically regulates plant response to concurrent biotic and abiotic stresses

**DOI:** 10.1080/15592324.2026.2670872

**Published:** 2026-05-11

**Authors:** Zheng Zhou, Peiwu Li, Daguang Cai

**Affiliations:** a Biomanufacturing Institute, Xianghu Laboratory, Hangzhou, China; b Department of Molecular Phytopathology and Biotechnology, Institute of Phytopathology, Christian-Albrechts University of Kiel, Kiel, Germany; c Key Laboratory of Biology and Genetic Improvement of Oil Crops, Ministry of Agriculture and Rural Affairs, Oil Crops Research Institute, Chinese Academy of Agricultural Sciences, Wuhan, China

**Keywords:** UV-B signaling, flg22, gene regulation, MicroRNA, transcription factor, yeast one-hybrid

## Abstract

Plants face various environmental stresses and have developed sophisticated adaptive mechanisms to cope with constraints. miR858a targets transcription factor (TF) MYB111 and has proven to be a key regulator in modulating plant responses to biotic and abiotic stresses. However, its transcriptional regulation and the underlying mechanisms remain unresolved. To this end, we performed the yeast one-hybrid (Y1H) assay using the miR858a promoter against an Arabidopsis transcription factor library. In this way, we identified 32 TFs that specifically interact with the miR858a promoter. By analysing expression profiles of candidate TFs under different stress conditions, mimicked by flg22, UV-B, and co-treatment of flg22/UV-B treatments, we prioritized candidate TFs, including five flg22-responsive TFs (ERF73, bZIP63, NF-YC6, AL7, and AIN1) and one UV-B-responsive TF, MYBD. Our data show that miR858a is antagonistically regulated by distinct stress-responsive TFs and by their interplay, thus highlighting, for the first time, a crucial role of miRNAs and their expression regulation in plant responses to diverse stress factors. These findings provide deeper insights into the mechanisms by which plants adapt to changing environments and offer valuable traits and approaches for breeding crops resistant to various concurrent stress factors.

## Introduction

Plants in natural environments are constantly challenged by multiple biotic and abiotic stresses that often occur simultaneously. To survive these diverse and dynamic conditions, plants need to integrate signals from different pathways and establish coordinated molecular responses. Ultraviolet-B (UV-B) radiation induces the biosynthesis of photoprotective phenolic metabolites such as flavonols and sinapoylmalate, which function as natural sunscreens.^1^^,^^2^ In contrast, pathogen-associated molecular patterns (PAMPs), exemplified by the bacterial flagellin-derived peptide flg22, activate pattern-triggered immunity (PTI) through binding to the pattern-recognition receptor FLS2, leading to extensive transcriptional reprogramming and the induction of antimicrobial secondary metabolites.^3^

More than four decades of research have shown that UV-B-induced flavonol pathway genes (FPGs) are strongly repressed when plants encounter pathogens or PAMPs. Because flavonoids and defense-related phenolics (e.g., lignin, scopoletin) share the precursor phenylalanine, this repression is considered a resource allocation strategy that prioritizes defense over photoprotection during immune activation.^4^ This antagonistic crosstalk between UV-B and PAMP signaling has been documented in multiple plant species.^5^^,^^6^ However, the molecular mechanisms that mediate this trade-off, particularly the regulatory components that integrate stress signals across transcriptional and post-transcriptional layers, remain insufficiently understood.

MicroRNAs (miRNAs) act as master regulators of gene expression by directing mRNA cleavage or translational inhibition, and they play essential roles in development, hormone signaling, and stress responses.^7^^-^^9^ Although miRNA function has been extensively studied, the transcriptional regulation of miRNA genes has only recently gained broader attention. Increasing evidence demonstrates that miRNA promoters are dynamically regulated by stress-responsive transcription factors, adding an additional regulatory layer that shapes miRNA output under complex environmental conditions.^10^^,^^11^ However, systematic identification of transcription factors directly binding to stress-responsive miRNA promoters remains limited, particularly in the context of concurrent stresses. Our previous studies demonstrated that miR858a, through post-transcriptional suppression of its target MYB111, functions as a critical regulatory layer in the crosstalk between UV-B and flg22 signaling.^12^^,^^13^ miR858a-mediated repression of MYB111 was shown to modulate FPG expression and to contribute to the rewiring of metabolic responses under simultaneous UV-B and PAMP challenges. These findings suggested that miR858a acts as a molecular node integrating dual environmental signals.

miR858a was selected as a model miRNA in this study. It directly targets the R2R3-MYB transcription factors, including MYB11, MYB12, and MYB111, which are central regulators of flavonol biosynthesis. Among these, MYB111 plays a dominant role in UV-B-induced flavonol accumulation and photoprotection. It has been demonstrated that miR858a cleaves the MYB111 transcripts, regulating its targets at post-transcriptional level rather than translational repression.^14^^,^^15^ Our previous studies demonstrated that miR858a expression is oppositely regulated by UV-B irradiation and flg22 treatment. It represents a regulatory node at the intersection of photoprotective and immune signaling pathways.^12^^,^^13^ However, the regulatory mechanisms of miR858a itself and the transcription factors (TFs) involved remain unresolved. In this study, we conducted a yeast one-hybrid (Y1H) assay with a near-genome-wide Arabidopsis TF library to identify TFs that interact with the miR858a promoter.

Here, we report a set of TFs that belong to diverse TF families with diverse functions. By combining Y1H screening with expression profiling, we identified several TFs that may interactively function as key nodes in the transcriptional control of miR858a in response to different stress factors. Our findings suggest a multi-layered regulatory network governing plant response to diverse stress factors offering opportunity to understand the mechanisms of action of miRNAs in regulating plant stress responses.

## Materials and methods

### Plant material and treatment

The *Arabidopsis thaliana* ecotype Columbia‐0 (Col‐0) was used in this study as wild‐type material. Plant treatment was performed as described by Zhou et al.^16^ Seeds were sown on Jiffy-7 peat pellets and cultivated under short-day conditions (8 h light/16 h dark). After five weeks of growth, plants were transferred to complete darkness for 48 h to uniformly suppress Chalcone synthase (CHS) transcript accumulation and minimize background flavonoid synthesis. Dark-adapted plants were then sprayed either with HPLC-grade water or with 1 mM flg22. Following a 1 h incubation in darkness to allow elicitor perception, plants were exposed to UV-B supplemented with visible light or to visible light alone for 4 h. This treatment regime generated four conditions: water/visible light (CK), flg22, UV-B, and flg22/UV-B cotreatment (F/U). For each biological replicate, nine whole seedlings were harvested, immediately flash-frozen in liquid nitrogen, and stored at –80 °C until further experiment.

### Yeast one-hybrid assay screening

#### Yeast strains and medium

Yeast strain YM4271 was used as the host strain to harbor the bait DNA. The components of yeast complete medium (yeast peptone dextrose adenine, YPAD) and the different synthetic dextrose (SD) media were purchased from Clontech (Takara, Beijing) and prepared according to the manufacturer's instructions.

#### Construction of a bait vector

The bait sequence, miR858a promoter fragment (1778 bp), was synthesized and cloned into the binding domain (BD) vector pHisi-1 to construct pHisi-1-miR858a, which plasmids were linearized by digestion with *AflII*, and then transformed into yeast strain YM4271. The colonies were selected on selective SD medium in the absence of histidine (SD/-His) and validated by PCR. The positive recombinant yeast carrying DB vector pHisi-1-miR858a was used for further experiments.

#### Utilization of *Arabidopsis* transcription factor library

As prey proteins interacting with the bait vector, a cDNA expression library of *Arabidopsis* transcription factors was developed and used to produce hybrids between the prey and a trans-activating domain.^17^ It was predicted that 1579 TFs comprising 62 TF families with known DNA-binding domains were present in the Arabidopsis genome after it was fully sequenced (Table S1), allowing the rapid, large-scale identification of protein—DNA interactions.

#### Self-activation test

The bait sequence/promoter itself may bind to endogenous TFs in yeast, resulting in basal expression of the downstream reporter gene, so self-activation is a common phenomenon and required before screening. Only when there are no endogenous TFs that can bind to the bait sequence or the binding interaction is rather weak, the basal expression of the reporter gene can be suppressed, and the Y1H can proceed. As a competitive inhibitor of the HIS3 gene product, a selective synthetic dextrose medium without histidine (SD/-His) with a series of different concentrations of 3-amino-1,2,4-triazole (3-AT) was applied to identify the minimal inhibitory concentration for the bait strain.

#### One-to-one mating-based Y1H screening

All 1579 TF strains were activated and grown overnight in a selective synthetic dextrose medium without tryptophan (SD/-Trp). Yeast strain carrying the bait was grown overnight in SD/-His medium at the same time. Equal volumes (20 µl) of TF strain and bait strain were transferred to a selective medium SD/-His/-Trp with a suitable defined concentration of 3-AT and incubated for 2–3 d at 30 °C. The positive colonies were selected and prepared for the second screening for further confirmation. The selected candidates identified from the initial screening were further confirmed by the second screening with the SD/-His/-Trp plate with the highest concentration of 3-AT. All colonies were validated by colony PCR and sequencing.

#### Point-to-point verification

All unique TF candidates screened above were selected for point-to-point verification on the SD/-His/-Trp plate with different concentrations of 3-AT. For this, a 100 µL stock culture of the TF library and 100 µL bait stock (miR858a promoter) were mixed and 2 mL YPDA liquid medium was added, and then placed in a 96-well plate for incubation overnight at 30 °C. The morphology of yeasts under the microscope was observed on the next day and a clover shape indicated a successful mating. In total, 10 µL of yeast solution was taken and a gradient dilution (10^−1^, 10^−2^, 10^−3^) was performed on an SD/-His/-Trp plate containing a suitably defined concentration of 3-AT for incubation for 2–3 d at 30 °C. The morphology of yeasts were documented.

### RNA isolation

Total RNA was isolated from Arabidopsis seedlings using TRIzol® reagent (Thermo Fisher Scientific, Germany) as described in^13^ and Zhou.^12^ Frozen tissue samples were first ground to a fine powder under liquid nitrogen and then transferred to prechilled tubes containing TRIzol® reagent. Following a 5 min incubation at room temperature, 200 µL chloroform was added. The mixture was vortexed thoroughly and centrifuged at 10,000 rpm for 15 min at 4 °C. The supernatant was collected, placed on ice for 30 min, and subsequently centrifuged at 12,000 rpm for 15 min at 4 °C to precipitate RNA. The resulting pellet was washed twice: first with 1 mL of 80% ethanol prepared with DEPC-treated water, and then with 1 mL of 100% ethanol. Each wash step included centrifugation at 12,000 rpm for 5 min at 4 °C, after which the supernatant was discarded. The washed RNA pellet was air-dried for 10 min and finally resuspended in 50 µL of DEPC-water. RNA integrity was verified by 1% agarose gel electrophoresis, and concentration was measured using a NanoVue Plus spectrophotometer (GE Healthcare Life Science). All downstream analyses were performed with RNA prepared from three independent biological replicates.

### Quantitative real-time RT–qPCR analysis

To evaluate the expression levels of upstream TF candidates, reverse transcription quantitative PCR (RT–qPCR) was performed. Complementary DNA (cDNA) was synthesized from 1 µg of total RNA using the RevertAid First Strand cDNA Synthesis Kit (Thermo Fisher Scientific, Germany) in a 20 µL reaction, incubated at 42 °C for 60 min. Approximately 50 ng of the resulting cDNA was used as template in subsequent RT‒qPCR assays, which were run with Maxima SYBR Green/ROX qPCR Master Mix (2X) (Thermo Fisher Scientific, Germany) on a real-time PCR system. Amplification conditions consisted of an initial denaturation at 95 °C for 10 min, followed by 40 cycles of 95 °C for 15 s, 59 °C for 30 s, and 72 °C for 30 s. A final melting curve analysis was performed from 65 to 95 °C to confirm amplicon specificity. Transcript levels of candidate TFs that interact with the miR858a promoter were normalized to the endogenous reference gene *Actin2*. Each treatment included three independent biological replicates, with two technical replicates per sample. Sequences and amplification efficiencies of primers used in RT‒qPCR are listed in Table S2.

### Identification of promoter motifs

The identification of motif for miR858a promoter was analysed by Plant Transcription Factor Database (PlantTFDB, http://planttfdb.gao-lab.org/)^18^ and Multiple Em for Motif Elicitation (MEME Suite, https://meme-suite.org/meme/).^19^

### Statistical analysis

In accordance with the methodology of^13^ and^12^ the experiments followed a completely randomized design. Each treatment was composed of three independent biological replicates. Data are plotted as mean ± SE in the figures. Multiple comparisons of statistical significance were carried out using a two-way ANOVA followed by Tukey's multiple comparison test according to Minitab software.^20^

## Result

### Screening the TFs interacting with the miR858a promoter

Yeast one-hybrid (Y1H) assays remain a powerful and widely used approach for identifying direct transcription factor–DNA interactions, and have recently been applied to dissect transcriptional regulation of stress-responsive genes and noncoding RNAs in plants.^21^^,^^22^ To identify transcription factors (TFs) that directly interact with the promoter of miR858a, the promoter fragment was synthesized and cloned into the pHisi-1 vector to generate the bait plasmid pHisi-1-miR858a. Prior to library screening, we determined the optimal concentration of 3-amino-1,2,4-triazole (3-AT) required to suppress self-activation of the bait strain. The inhibitory assay revealed that 60 mM 3-AT was sufficient to reduce background growth, whereas concentrations above 100 mM completely eliminated self-activation (Figure S1A), allowing reliable screening.

Using the Arabidopsis cDNA library, initial screening on SD/-His/-Trp plates supplemented with 60 and 100 mM 3-AT yielded 86 colonies capable of activating the *HIS3* reporter (Figure S1B). These putative positives were subjected to a second round of screening, followed by colony PCR and sequencing validation, ultimately identifying 43 unique TF candidates that interacted with the miR858a promoter (Figure S1C; Table S3).

**Figure 1.**
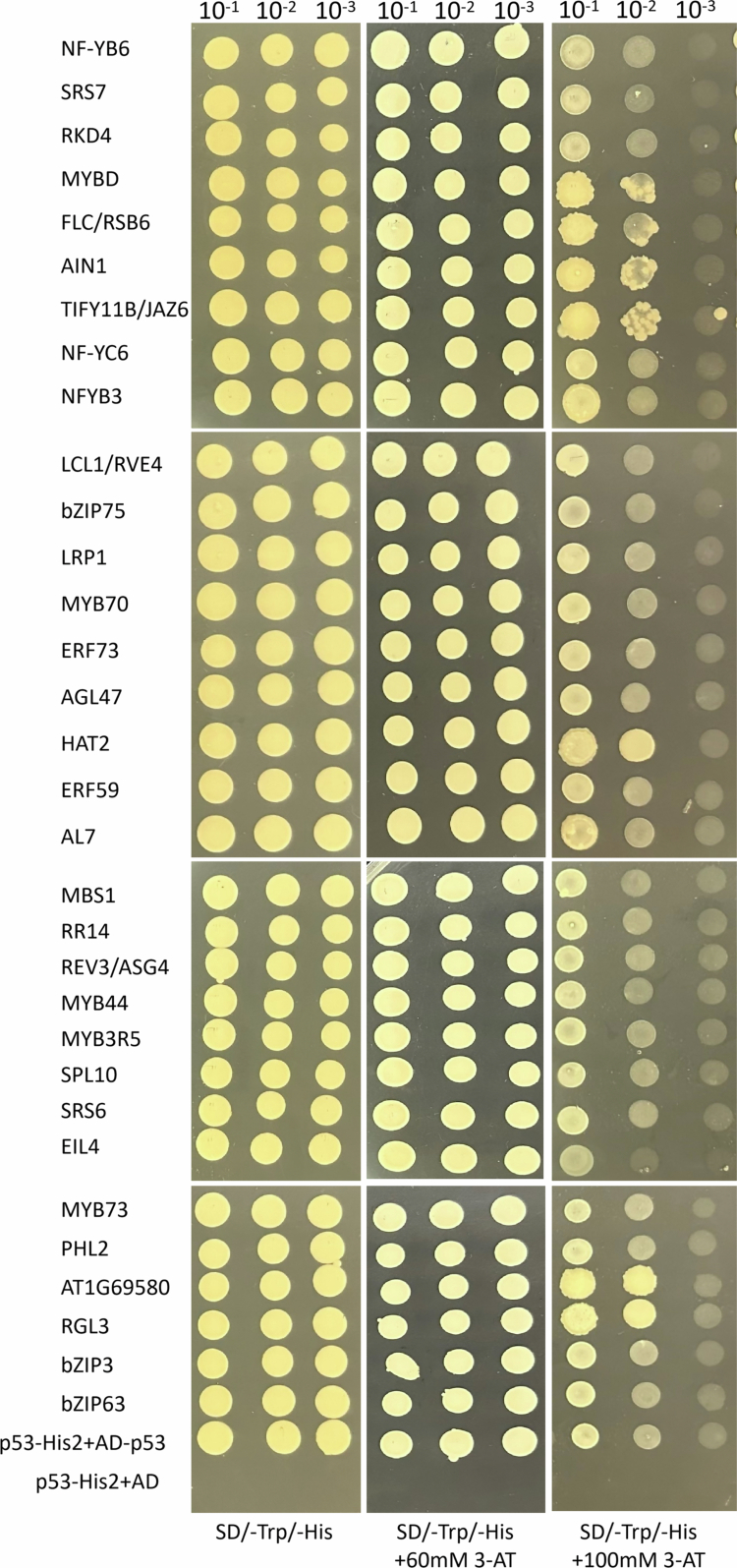
Point-to-point verification of mating-based screening. Screening with the miR858a promoter as bait in a Y1H. All 32 positive clones were diluted to 10^−1^, 10^−2^, and 10^−3^, and then plotted on nonselective SD/-Trp/-His and selective SD/-Trp/-His supplemented with 60 and 100 mM 3-AT plates. Yeast growth on the non-selective medium SD/-Trp/-His showed mating efficiency.

To validate these interactions, point-to-point yeast one-hybrid assays were performed for all candidates after removing duplicates (e.g., NF-YB3, MYB70, RR14, MYB44) and two proteins lacking reliable annotations. Yeast cells harboring each TF clone exhibited robust growth on selective medium containing either 60 or 100 mM 3-AT, confirming specific interaction with the miR858a promoter (Figure 1). In total, 32 TFs showed reproducible promoter binding in the validation assay. Functional enrichment analysis revealed that these TFs are predominantly associated with responses to abiotic and biotic stresses, external and light stimuli, and secondary metabolic processes, suggesting that miR858a is tightly embedded in multiple stress-responsive and metabolic regulatory pathways.

### Prediction of regulatory motifs recognized by miR858a-binding TFs

To determine the potential motifs mediating TF binding on the miR858a promoter, we predicted motif preferences for all 32 validated TFs using PlantTFDB and MEME Suite. The TF candidates belong to diverse families, including MYB, MYB-related, AP2, bZIP, HD-ZIP, NF-Y, MADS, GRAS, SRS, PLATZ, and SBP, which collectively recognize a broad range of motifs such as the CTCCTCCTC, CGGTGG, and CCACACGC motifs, and several family-specific motifs (Table 1). Motif scanning further revealed a non-random spatial distribution of predicted TF-binding elements along the miR858a promoter (Figure S2). ERF/AP2-associated CTCCTCCTC motifs were mainly enriched in the distal promoter region (approximately −500 to −1,200 bp), whereas MYB-related CGGTGG elements were preferentially located near the transcription start site (TSS). Notably, several TF families possess multiple predicted binding sites within the promoter, suggesting potential combinatorial or competitive promoter occupancy. The presence of multiple light-, stress-, and hormone-responsive motifs within the miR858a promoter is consistent with the complex regulatory landscape indicated by the TF family composition. These motif-TF associations provide a mechanistic basis for understanding how miR858a integrates diverse upstream signaling inputs.

**Table 1.** The predicted motifs of TF candidates interacting with the miR858a promoter.

Gene ID	TF family	Motif sequence	Symbols
AT1G01520	MYB-related	CGAAGSCAMGCAC	ASG4, altered seed germination 4, REV3, REVEILLE 3
AT1G06160	AP2-EREBP	CTCCTCCTC	ORA59, octadecanoid-responsive Arabidopsis AP2/ERF 59, ERF59, ethylene responsive factor 59
AT1G14510	Alfin	–	AL7, alfin-like 7, ATAL7
AT1G19790	SRS	ACGGTGGWSATCWGAMGATGGA	SRS7, SHI-related sequence 7, SPRI2, stigmatic privacy 2
AT1G21000	PLATZ	–	PLATZ1, AIN1, ABA-induced expression 1
AT1G27370	SBP	GGYACACTGC	SPL10, squamosa promoter binding protein-like 10
AT1G69580	GARP-G2-like	GGTGACAC	PHL8, G2-like family protein
AT1G70000	MYB-related	GGYGGGG	MYB-related family protein
AT1G72360	AP2-EREBP	GGGAGG	AtERF73, ERF073, ERF73, HRE1, ERF family protein
AT1G72450	OB24	–	JAZ6, jasmonate-zim-domain protein 6, TIFY11B, TIFY domain protein 11B
AT2G01760	GARP-ARR-B	GTCACSCG	ARR14, RR14, response regulator 14
AT2G23290	MYB	CTCCTCCTC	MYB70, AtMYB70, myb domain protein 70
AT3G02790	zinc finger	–	MBS1, methylene blue sensitivity 1
AT3G24120	GARP-G2-like	CGGTGGAC	PHL2, PHR1-LIKE 2
AT3G54430	SRS	CGGTGG	SRS6, SHI-related sequence 6
AT4G14540	NF-YB	CACCSGTGAG	NF-YB3, nuclear factor Y, subunit B3
AT4G37260	MYB	CGGTGG	MYB73, MYB domain protein 73, ATMYB73, NRM1A, *N* required MYB-like transcription factor 1 A
AT5G02320	MYB	CGGTGG	MYB3R-5, MYB domain protein 3R-5, ATMYB3R5, Arabidopsis thaliana MYB domain protein 3R5
AT5G02840	MYB-related	GTGGGG	LCL1, LHY/CCA1-like 1, RVE4, REVEILLE 4
AT5G08141	bZIP	CGGATGSG	bZIP75, basic leucine-zipper 75, AtbZIP75, basic leucine-zipper 75
AT5G10120	EIL	GGTGGGGARAACWWGGAG	EIL4, EIL family protein
AT5G10140	MADS	GGCAAGC	FLC, flowering locus C, FLF, flowering locus F, AGL25, Agamous-like 25, RSB6, reduced stem branching 6
AT5G12330	SRS	CGAGYCASCCG	LRP1, lateral root primordium 1
AT5G15830	bZIP	CGAAGSCAAGC	AtbZIP3, basic leucine-zipper 3, bZIP3, basic leucine-zipper 3
AT5G17490	GRAS	CGGTGGAC	RGL3, RGA-like protein 3, AtRGL3
AT5G28770	bZIP	CCACACGC	BZO2H3, BZIP63, AtbZIP63, Arabidopsis thaliana basic leucine zipper 63
AT5G47370	HD-ZIP	CCACACGCWCG	HAT2, Homeobox-leucine zipper protein 4 (HB-4)/HD-ZIP protein
AT5G47670	NF-YB	CACASGCMCGWAGGC	NF-YB6, nuclear factor Y, subunit B6, L1L, LEC1-like
AT5G50480	NF-YC	CCACACGC	NF-YC6, nuclear factor Y, subunit C6
AT5G53040	Nin-like	CGGTGG	RKD4, RWP-RK domain-containing 4, GRD, GROUNDED
AT5G55690	MADS	GGCCCRAGC	AGL47, Agamous-like 47
AT5G67300	MYB	GGTGGG	MYBR1, MYB domain protein R1, ATMYBR1, ATMYB44, Arabidopsis thaliana MYB domain protein 44, NRM1B, *N* required MYB-like transcription factor 1B, MYB44

### Differential expression of miR858a-regulating TFs under flg22 and UV-B treatments

**Figure 2.**
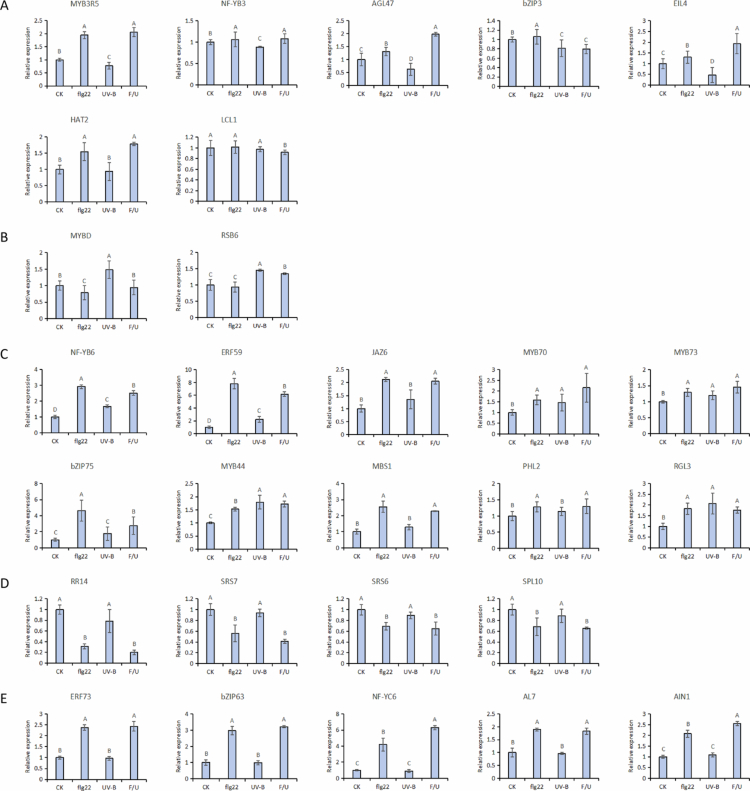
Relative expression analysis of transcription factors regulating miR858a in response to flg22, UV-B, and co-treatment F/U. (A) TF candidates upregulated by flg22 but downregulated by UV-B. (B) TF candidates downregulated by flg22 but upregulated by UV-B. (C) TF candidates upregulated by both flg22 and UV-B. (D) TF candidates were downregulated by both flg22 and UV-B. (E) TF candidates specifically respond to flg22. The relative expression level is based on the comparative Ct method.^23^ Data were obtained from three independent biological samples and represent the mean ± SE. Normalization of the expression levels was carried out using *Actin2* as an internal reference gene. Statistically significant differences between the mock control levels (CK) and treatment transcripts were determined by a two-way ANOVA followed by Tukey’s multiple comparison test.

To determine how biotic and abiotic stresses regulate these TFs, we examined the transcript abundance of all 32 candidates under flg22, UV-B, and combined flg22/UV-B (F/U) treatments using RT–qPCR. The TFs displayed distinct and often contrasting transcriptional patterns, illustrating a highly dynamic regulatory network upstream of miR858a. A group of TFs exhibited antagonistic regulation, being strongly induced by flg22 but repressed by UV-B. This category includes MYB3R5, NF-YB3, AGL47, bZIP63, EIL4, and HAT2 (Figure 2A). These TFs likely contribute to defense-induced activation of miR858a, while UV-B may counteract this activation through transcriptional suppression. Conversely, MYBD showed the opposite pattern, being suppressed by flg22 but induced by UV-B. RSB6 displayed relatively higher expression under UV-B treatment compared with flg22 treatment (Figure 2B). Together, the antagonistic TF groups support the hypothesis that flg22- and UV-B-mediated cues converge on miR858a through opposing regulatory modules. Several TFs responded synergistically to both flg22 and UV-B, including NF-YB6, ERF59, JAZ6, MYB70, MYB73, bZIP75, MYB44, MBS1, PHL2, and RGL3 (Figure 2C). The concurrent induction of these regulators suggests the presence of shared signaling nodes or reinforcing pathways that collectively enhance miR858a expression under multiple stresses. Four TFs, namely RR14, SRS7, SRS6, and SPL10, were consistently repressed by both flg22 and UV-B (Figure 2D), implying that they may act as negative regulators whose suppression could relieve inhibition of miR858a transcription. In addition, several TFs appeared to be specifically responsive to biotic signaling, with notable flg22-induced upregulation observed for ERF73, bZIP63, NF-YC6, AL7, and AIN1 (Figure 2E). The selective responsiveness of these TFs highlights the specificity of microbe-associated molecular pattern (MAMP) signaling in shaping miR858a regulation. Collectively, these expression patterns demonstrate that miR858a is embedded within a complex transcriptional network that integrates both UV-B-mediated abiotic stress and flg22-induced immune signals. The diverse and often contrasting regulatory behaviours of upstream TFs provide strong evidence that miR858a functions as a convergence point of stress-responsive signaling modules, bridging metabolic rewiring, growth regulation, and innate immunity.

## Discussion

Recent conceptual frameworks propose that miRNAs function as regulatory hubs that enhance network robustness by buffering transcriptional noise and integrating multiple upstream signals.^24^^,^^25^ In line with this view, our identification of diverse stress-responsive transcription factors upstream of miR858a supports a model in which miR858a acts as a central integrator of UV-B and immune signaling, dynamically adjusting flavonoid biosynthesis and defense prioritization under combined stress conditions.

### miR858a as a central regulatory node integrating biotic and abiotic stimuli

Our study establishes miR858a as a key transcriptional node that integrates environmental signals through a diverse set of upstream transcription factors. The identification of 32 TFs interacting with the miR858a promoter, including MYB, ERF, bZIP, NF-Y, MADS-box, SBP, HD-ZIP, GRAS, SRS, PLATZ, and other families, highlights the extensive regulatory convergence upon this single miRNA locus. Such diversity is notable given that individual miRNAs are often regulated by only a handful of TFs, and it suggests that miR858a exerts broad influence across stress responses, metabolic adjustment, and developmental transitions. The enrichment of light-, hormone-, immune-, and secondary metabolism-related motifs further supports the concept that miR858a is embedded within a multilayered regulatory architecture.

The transcript profiling of these TFs under flg22 and UV-B, two representative biotic and abiotic stresses, revealed an intricate pattern of antagonistic, synergistic, or stimulus-specific regulation. This complexity underscores that miR858a is not simply stress-inducible or repressible; rather, its expression is shaped by competing and reinforcing signals that collectively modulate plant energy allocation between defense, growth, and photoprotection.

### Convergence and antagonism between flg22-triggered immunity and UV-B signaling upstream of miR858a

Flg22 and UV-B represent distinct upstream pathways, pattern-triggered immunity (PTI) and photomorphogenic/light stress signaling, respectively. Yet many TFs identified here showed co-regulation by both stimuli, indicating shared transcriptional nodes that feed into miR858a.^4^^,^^13^

A subset of TFs, including MYB3R5, NF-YB3, AGL47, bZIP63, EIL4, and HAT2, was strongly induced by flg22 but repressed by UV-B. These antagonistic dynamics suggest molecular competition between immune activation and UV-B–induced growth restriction. Because miR858a is known to suppress MYB11/12/111-mediated flavonoid biosynthesis, flg22-induced TFs may act to elevate miR858a and thereby redirect metabolic flux away from flavonoid production toward defense-related pathways. Conversely, the UV-B response requires elevated flavonoid accumulation to enhance epidermal shielding and reactive oxygen species (ROS) quenching; thus, UV-B repression of these TFs could release miR858a inhibition and promote MYB111-driven flavonoid biosynthesis.

This reciprocal regulation aligns with the classical “growth–defense trade-off”^26^ and suggests that miR858a operates at a critical decision-making point where PTI-driven resource mobilization conflicts with UV-B–driven photoprotection. Conversely, MYBD and RSB6, which were suppressed by flg22 but induced by UV-B, may function as abiotic-stress-specific repressors or activators of miR858a, further modulating metabolic prioritization. Such opposing transcriptional modules create a tunable rheostat system rather than a binary switch, ensuring that miR858a integrates stress intensities and temporal contexts.

### Synergistic induction of miR858a regulators suggests shared transcriptional modules in dual-stress environments

In addition to antagonistic TFs, we identified a cluster of factors, including NF-YB6, ERF59, JAZ6, MYB70, MYB73, bZIP75, MYB44, MBS1, PHL2, and RGL3, which were upregulated by both flg22 and UV-B. The concurrent induction of these TFs implies the presence of shared signaling nodes activated by multiple stress cues, potentially including ROS waves, MAPK cascades, JA/ET crosstalk, or chromatin modifications triggered by general stress signatures.

These TFs may represent the “core stress-responsive module” regulating miR858a, functioning as integrators of generalized danger or energy deprivation signals. Induction of miR858a through these TFs under combined stresses may allow plants to suppress unnecessary specialist metabolic pathways and maintain robustness under converging environmental challenges.

The repression of TFs such as RR14, SRS7, SRS6, and SPL10 by both stimuli suggests a complementary mechanism whereby negative regulators are silenced to further fine-tune miR858a expression.

### miR858a balances flavonoid biosynthesis and PTI activation through MYB111

Recent studies highlight that secondary metabolites such as flavonoids not only function as protective compounds but also actively modulate immune signaling by shaping cellular redox status and hormone signaling pathways.^27^^-^^29^ Thus, miR858a-mediated modulation of MYB111-dependent flavonoid biosynthesis may represent a mechanism by which plants fine-tune immune responsiveness under fluctuating environmental constraints.

The regulatory network uncovered here provides a mechanistic explanation for how miR858a coordinates flavonoid biosynthesis and immune activation. miR858a is a well-characterized suppressor of the R2R3-MYB activators of phenylpropanoid and flavonoid pathways, chiefly MYB11, MYB12, and MYB111. Among them, MYB111 plays a central role in the production of UV-B-absorbing flavonols, compounds essential for photoprotection and ROS detoxification.^30^^,^^31^

Under UV-B, our data suggest that repression of flg22-induced TFs and activation of UV-B-specific regulators collectively suppress miR858a expression, thereby enhancing MYB111 activity and flavonoid accumulation. This allows plants to redirect resources toward UV-shielding pigments. Conversely, during flg22-triggered immunity, the induction of TFs promoting miR858a expression may attenuate MYB111 activity, reducing allocation to flavonoid biosynthesis and freeing metabolic resources for PTI-associated defense biosynthesis. Such a mechanism would align with the observation that flavonoids can dampen immune signaling through antioxidant activity that interferes with ROS-mediated defense amplification. Thus, transient suppression of flavonoid biosynthesis via miR858a may serve to ensure robust PTI signaling. Collectively, these findings support a regulatory model in which miR858a functions as a molecular "balancing valve" that modulates MYB111-dependent flavonoid accumulation in accordance with environmental demands.

### A proposed mechanistic model for TF–miR858a–MYB111 coordination of stress adaptation

**Figure 3.**
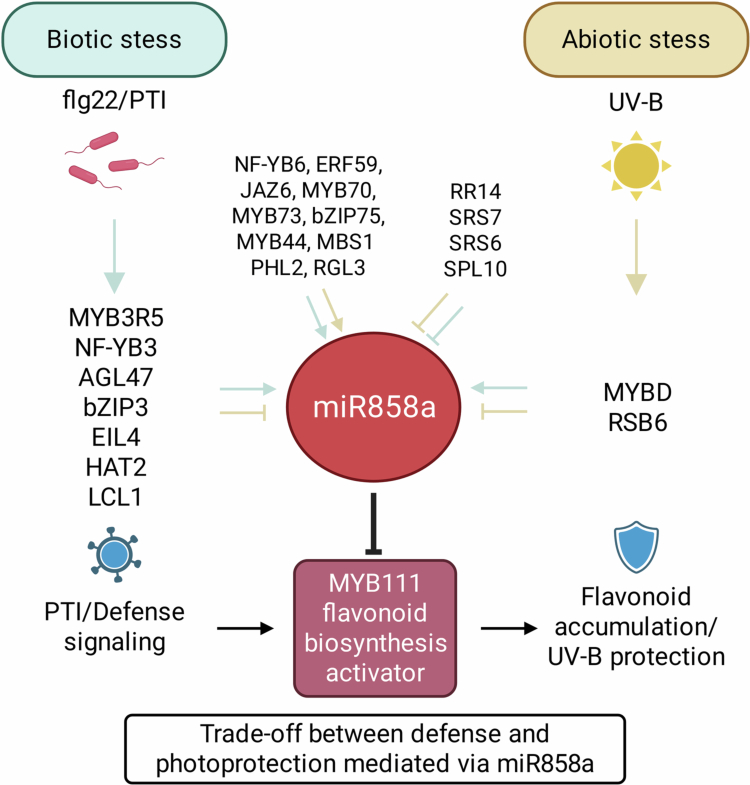
Proposed regulatory network of miR858a in Arabidopsis integrating biotic (flg22) and abiotic (UV-B) signals. TFs responding to flg22 or UV-B activate or repress miR858a, which in turn negatively regulates MYB111 to modulate flavonoid biosynthesis. Shared TFs and negative regulators create a tunable system that balances photoprotection and immune responses.

Based on our results, we propose that diverse TF families bind to distinct motifs on the miR858a promoter, forming a multilayered regulatory module integrating immune signals, light cues, hormonal pathways, and metabolic status (Figure 3). While Y1H assays provide direct evidence for TF–promoter interaction, the regulatory relationships inferred from expression profiling should be interpreted as a working model. Flg22-induced TFs activate miR858a and lead to suppression of MYB111 and flavonoid biosynthesis, thereby promoting PTI signaling by allowing ROS-based defense amplification. While UV-B-induced TFs repress miR858a, release MYB111, and enhance flavonoid production, enabling stronger photoprotection and ROS buffering. Moreover, synergistic TFs act as universal stress modulators, providing a backbone for miR858a expression under combined or fluctuating stress environments. The directional outcome of miR858a expression depends on quantitative TF competition, promoter occupancy, and temporal dynamics, forming an environmentally responsive regulatory circuit. Collectively, this framework demonstrates that miR858a acts as a molecular integrator that resolves conflicting metabolic demands between UV-B photoprotection and immune activation, allowing plants to achieve flexible and optimized stress adaptation.

While this study systematically identifies transcription factors binding to the miR858a promoter, several limitations should be acknowledged. First, Y1H assays were conducted in a heterologous yeast system, which may not fully recapitulate chromatin context in planta. Second, promoter occupancy dynamics under native stress conditions were not directly measured. Third, quantitative competition among antagonistically regulated TFs remains unresolved. Future studies employing chromatin immunoprecipitation, promoter-reporter assays, and genetic mutant analysis will be required to dissect the functional hierarchy and temporal dynamics of TF–miR858a interactions in vivo.

## Conclusion

In this study, we demonstrate that miR858a is transcriptionally regulated by a set of stress-responsive transcription factors, which control plant response to biotic and abiotic stress. These TFs exhibit antagonistic and synergistic interaction modules on the miR858a promoter, with their expression profiles distinguishing between abiotic and biotic stresses. These data corroborate miR858a’s role as a hub miRNA in regulating plant response to various stresses via balancing flavonoid biosynthesis and immune responses. These findings substantially extend our understanding of how miRNAs work to reprogram plant responses to cope with diverse stress factors. The proposed model provides a basis for further research, facilitating a deep understanding of the mechanisms of action and developing new approaches to enhance crop resilience through editing miRNA-centered regulatory networks in crops.

## Supplementary Material

Supplementary Material Supplementary_Table_(new)_07_May_2026_12_58_AU.xlsx

Supplementary_Figure_caption Supplementary_Figure_caption.

Figure S1.tif Figure S1.tif

Figure S2.tif Figure S2.tif
