# Supplementary material for: MicroRNA858a antagonistically regulates plant response to concurrent biotic and abiotic stresses
Source: Plant Signal Behav. 2026 May 11;21(1):2670872. doi: 10.1080/15592324.2026.2670872 (PMC13170379; doi:10.1080/15592324.2026.2670872)
Supplement: Supplementary_Figure_caption [file KPSB_A_2670872_SM5542.docx]

**Figure S1** Results of self-activation on SD/-His supplemented with different concentration of 0, 3, 5, 7, 10, 30, 45, 60, 80 and 100mM 3-AT (A), initial screening on none-selective SD/-Trp/-His and selective SD/-Trp/-His supplemented with 60 and 100mM 3-AT plates (B) and second screening on selective SD/-Trp/-His supplemented with 100mM 3-AT plates.

**Figure S2** Schematic distribution of predicted transcription factor binding sites along the miR858a promoter.
