# Supplementary figures and images for: MicroRNA858a antagonistically regulates plant response to concurrent biotic and abiotic stresses
Source: Plant Signal Behav. 2026 May 11;21(1):2670872. doi: 10.1080/15592324.2026.2670872 (PMC13170379; doi:10.1080/15592324.2026.2670872)

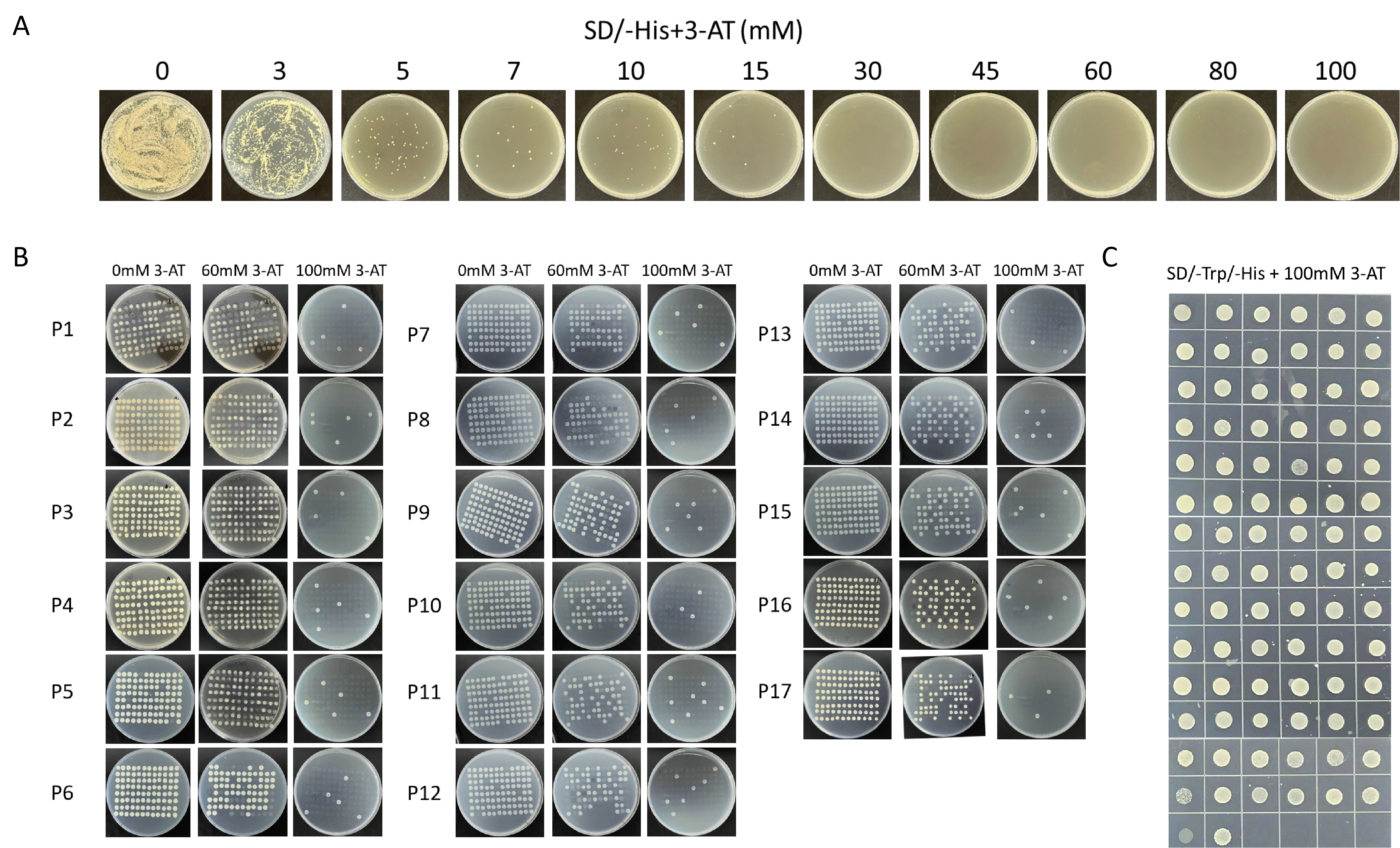

Supplement: Figure S1.tif [file KPSB_A_2670872_SM1531.tif]

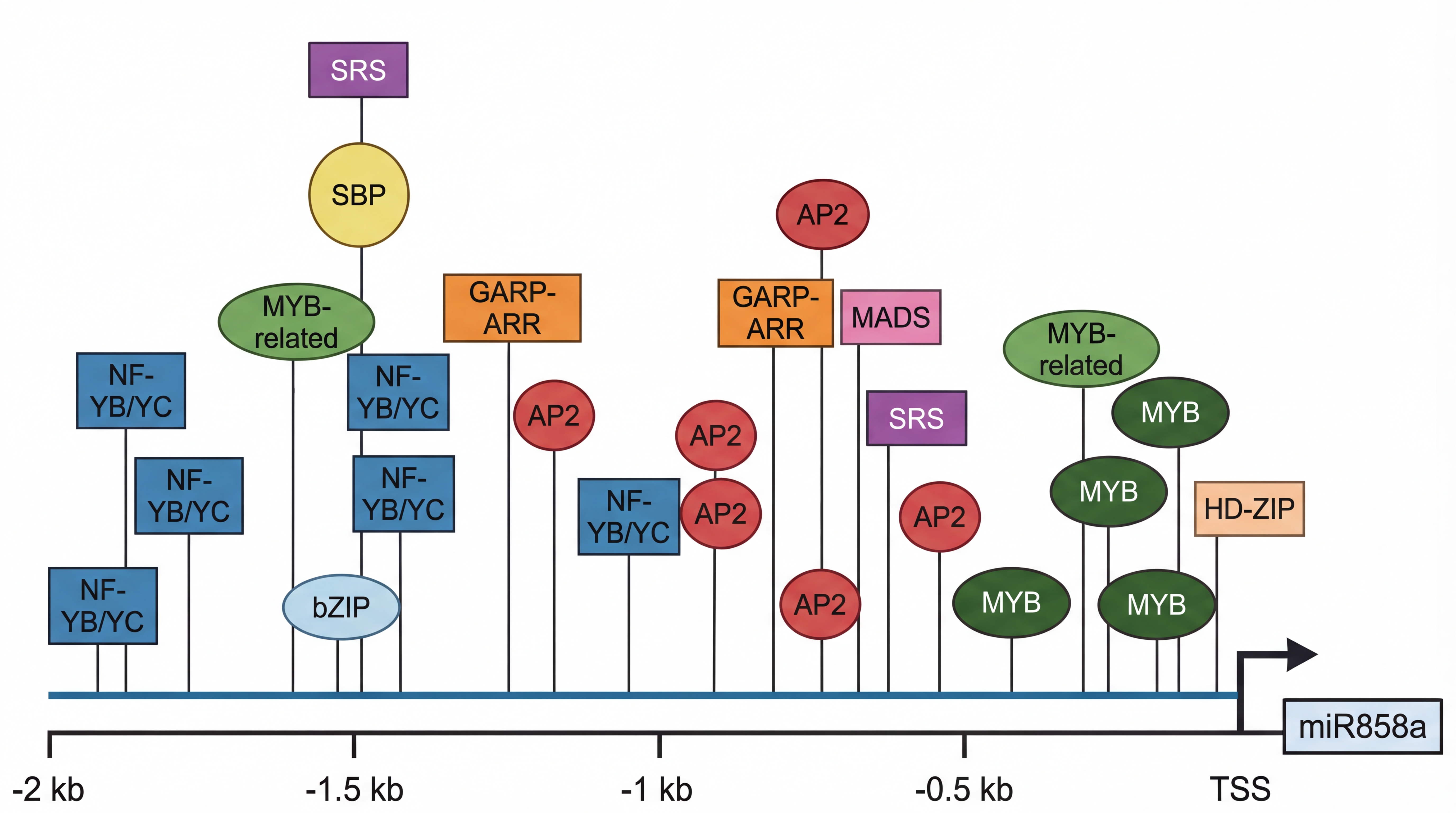

Supplement: Figure S2.tif [file KPSB_A_2670872_SM1530.jpg]
